# Cathelicidin-OA1, a novel antioxidant peptide identified from an amphibian, accelerates skin wound healing

**DOI:** 10.1038/s41598-018-19486-9

**Published:** 2018-01-17

**Authors:** Xiaoqing Cao, Ying Wang, Chunyun Wu, Xiaojie Li, Zhe Fu, Meifeng Yang, Wenxin Bian, Siyuan Wang, Yongli Song, Jing Tang, Xinwang Yang

**Affiliations:** 1 Department of Pathology, Faculty of Basic Medical Science, Kunming Medical University, Kunming, 650500 Yunnan China; 2 Key Laboratory of Chemistry in Ethnic Medicine Resource, State Ethnic Affairs Commission & Ministry of Education, School of Ethnomedicine and Ethnopharmacy, Yunnan Minzu University, Kunming, 650500 Yunnan China; 3 Department of Anatomy and Histology & Embryology, Faculty of Basic Medical Science, Kunming Medical University, Kunming, 650500 Yunnan China; 4 Department of Biochemistry and Molecular Biology, Faculty of Basic Medical Science, Kunming Medical University, Kunming, 650500 Yunnan China

## Abstract

Cathelicidins play pivotal roles in host defense. The discovery of novel cathelicidins is important research; however, despite the identification of many cathelicidins in vertebrates, few have been reported in amphibians. Here we identified a novel cathelicidin (named cathelicidin-OA1) from the skin of an amphibian species, *Odorrana andersonii*. Produced by posttranslational processing of a 198-residue prepropeptide, cathelicidin-OA1 presented an amino acid sequence of ‘IGRDPTWSHLAASCLKCIFDDLPKTHN′ and a molecular mass of 3038.5 Da. Functional analysis showed that, unlike other cathelicidins, cathelicidin-OA1 demonstrated no direct microbe-killing, acute toxicity and hemolytic activity, but did exhibit antioxidant activity. Importantly, cathelicidin-OA1 accelerated wound healing against human keratinocytes (HaCaT) and skin fibroblasts (HSF) in both time- and dose-dependent manners. Notably, cathelicidin-OA1 also showed wound-healing promotion in a mouse model with full-thickness skin wounds, accelerating re-epithelialization and granulation tissue formation by enhancing the recruitment of macrophages to the wound site, inducing HaCaT cell proliferation and HSF cell migration. This is the first cathelicidin identified from an amphibian that shows potent wound-healing activity. These results will help in the development of new types of wound-healing agents and in our understanding of the biological functions of cathelicidins.

## Introduction

Cathelicidins, which belong to a group of cationic peptides with amphipathic properties, play critical roles in host defense^1^. Cathelicidins show broad-spectrum antimicrobial properties and are characterized by a N-terminus signal peptide and highly conserved cathelin domain, followed by a C-terminal mature peptide featured by remarkable structural diversity^2^. Apart from their direct antimicrobial activities, cathelicidins can also trigger specific defense responses, such as antioxidant, immunomodulatory, and hemolytic activities as well as inhibition of apoptosis^3^. About 30 cathelicidin family members have been identified in mammalian species, including humans, cattle, buffalo, horses, pigs, sheep, goats, deer, chickens, fish, and snakes^4^. Although more than 7,000 amphibian species are currently described in the AmphibiaWeb database, with nearly 90% being frogs (https://en.wikipedia.org/wiki/Amphibian)^5^, only seven frog cathelicidins have been reported thus far. These include cathelicidin-AL from *Amolops loloensis*^6^, cathelicidin-RC1 and cathelicidin-RC2 from *Rana catesbeiana*^7^, Lf-CATH-1 and -2 from *Limnonectes fragilis*^8^, cathelicidin-PY from *Paa yunnanensis*^9^ and cathelicidin-PP from *Polypedates puerensis*^5^. Thus, considerable information remains unavailable but urgently required for understanding the structure and function of amphibian cathelicidins.

Promotion of wound healing is a key goal of medical treatment. As the largest organ of the body, skin is a crucial barrier between the internal body and outside environment and performs pivotal physiological functions, such as perspiration and heat and pain sensation^10^. Once the skin is damaged, the resulting disruption between the inner body and outer surroundings can lead to poor nutrition, ill health, and death. Skin wounds place severe physical and financial burdens upon human society^11^, and thus there is an urgent need to explore effective remedies for wound healing^12^. Traditional wound healing drugs hold many disadvantages, such as high cost, low activity, and easily produced hyperplastic scars^13^. Compared with such drugs, bioactive peptides, which are distinguished from proteins and contain approximately 50 or fewer amino acids, exhibit high activity, specificity, and stability. These advantages have aroused considerable global interest in their identification, including antimicrobial peptides (AMPs), analgesic peptides, and antineoplastic polypeptides^14^. However, peptides exhibiting potent wound-healing promoting potency have been reported rarely^15^.

Amphibians live in harsh environments, with their exposed skin making them vulnerable to microorganisms, ultraviolet radiation and injury^16^. To combat these factors, they have evolved a unique and highly effective polypeptide defense system in their skin^17^. Amphibian skin have been proven to secrete diversity of small bioactive peptides with diverse pharmacological bioactivities, including antimicrobial, antioxidant, and immunomodulatory activities^18^. Previous research has demonstrated that amphibian skin can repair itself quickly after damage, and thus has potential in wound healing drug development^19,20^. However, research on wound-healing promoting peptides, especially those found in amphibian species, is still in its infancy.

Our previous research revealed very high AMP abundance and diversity in *O*. *andersonii*, indicating that many unknown bioactive peptides remain to be identified^17^. In the present study, a novel cathelicidin-like peptide, cathelicidin-OA1, was identified from *O. andersonii* skin secretions. Our results demonstrated that this novel peptide could promote wound healing at the cellular level and in an animal model. The underlying mechanisms of wound-healing promotion were also explored. Results indicated that HaCaT cell proliferation, HSF cell migration, and significant pro-inflammatory effects were likely involved in the healing of wounds induced by cathelicidin-OA1. Furthermore, cathelicidin-OA1 accelerated re-epithelialization and granulation tissue formation of the skin wounds in the mouse model by enhancing the recruitment of macrophages to the wound site. Importantly, cathelicidin-OA1 is the first cathelicidin from a frog species to demonstrate wound healing activity.

## Results

### Peptide purification

**Figure 1:**
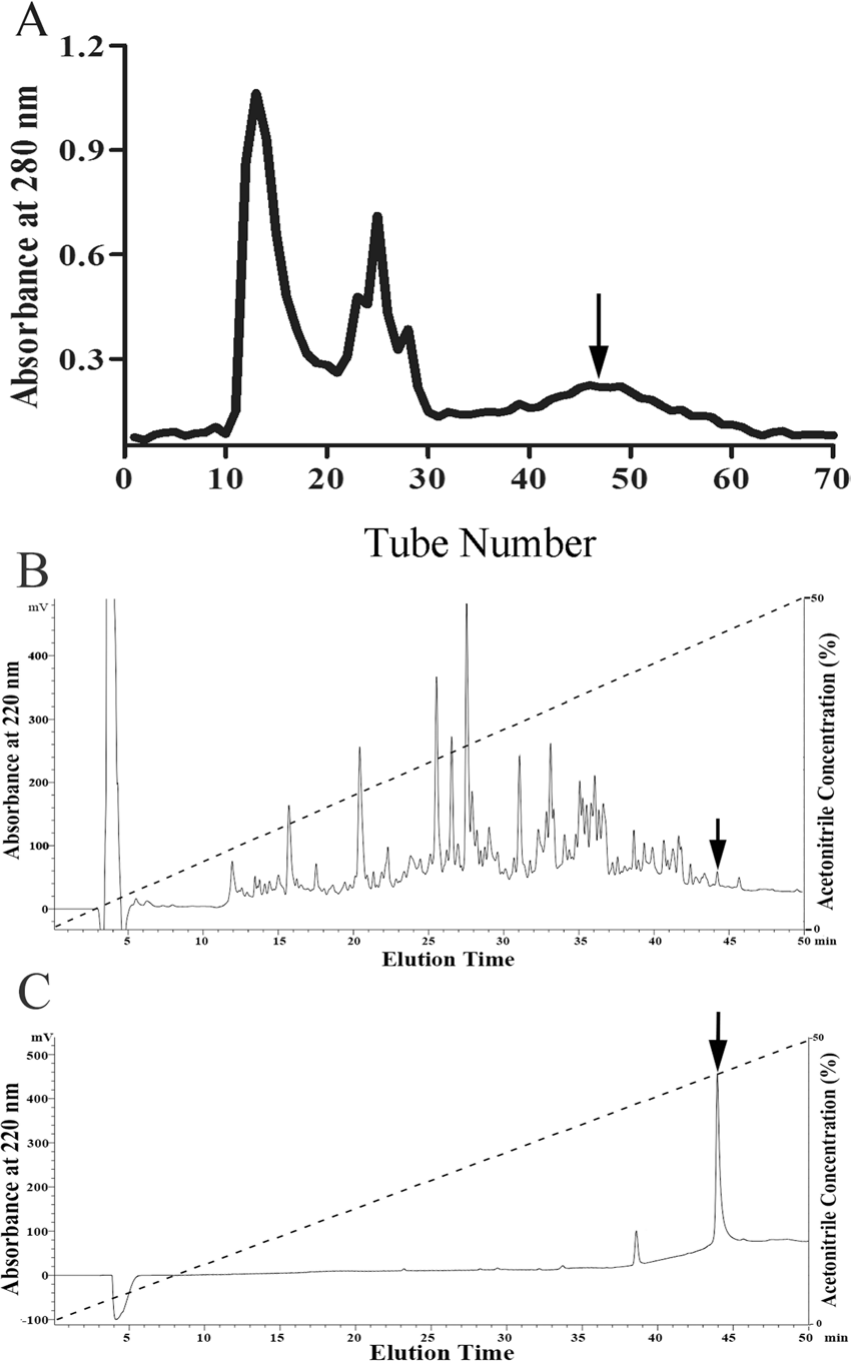
Peptide purification procedure from *O. andersonii* skin secretions. Skin secretions of *O. andersonii* were separated by a Sephadex G-75 gel column, with the peak exhibiting ABTS^+^ free radical scavenging activity indicated by an arrow. (**B**) Eluted peak in (**A**) showing ABTS^+^ free radical scavenging activity was collected for further purification using a RP-HPLC column. (**C**) Sample exhibiting ABTS^+^ free radical scavenging activity (indicated by arrow in (**B**)) was collected for further purification using a RP-HPLC column. A peptide with ABTS^+^ free radical scavenging activity was obtained (indicated by an arrow in C).

Through gel filtration, *O. andersonii* skin secretions showed several peaks (Fig. 1A). Gel filtration samples were collected every 10 min and their ABTS^+^ scavenging activities were tested. The fractions with obvious antioxidant activity (indicated by an arrow, Fig. 1A) were further purified by C18 RP-HPLC. As shown in Fig. 1B, more than 30 peaks were obtained, with one showing the required activity (indicated by an arrow, Fig. 1B). This peak was further purified by HPLC, and one peak with an ideal shape and identical elution time (indicated by an arrow in Fig. 1C) as that of the former HPLC procedure was obtained. This sample exhibited ABTS^+^ free radical scavenging activity (data not shown) and its primary structure was therefore determined.

### Primary structure of cathelicidin-OA1

**Figure 2:**
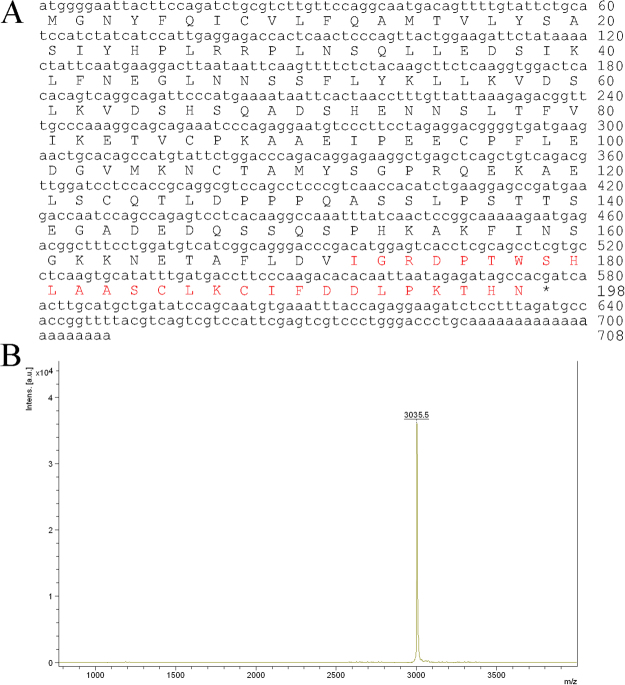
Primary structure of cathelicidin-OA1. (**A**) cDNA sequence of cathelicidin-OA1. The mature peptide (‘IGRDPTWSHLAASCLKCIFDDLPKTHN′) was 27 amino acid residues in length (red), and was produced by posttranslational processing of a 198-residue prepropeptide. (**B**) Observed molecular mass of cathelicidin-OA1 (indicated by an arrow in Fig. 1C).

**Figure 3:**
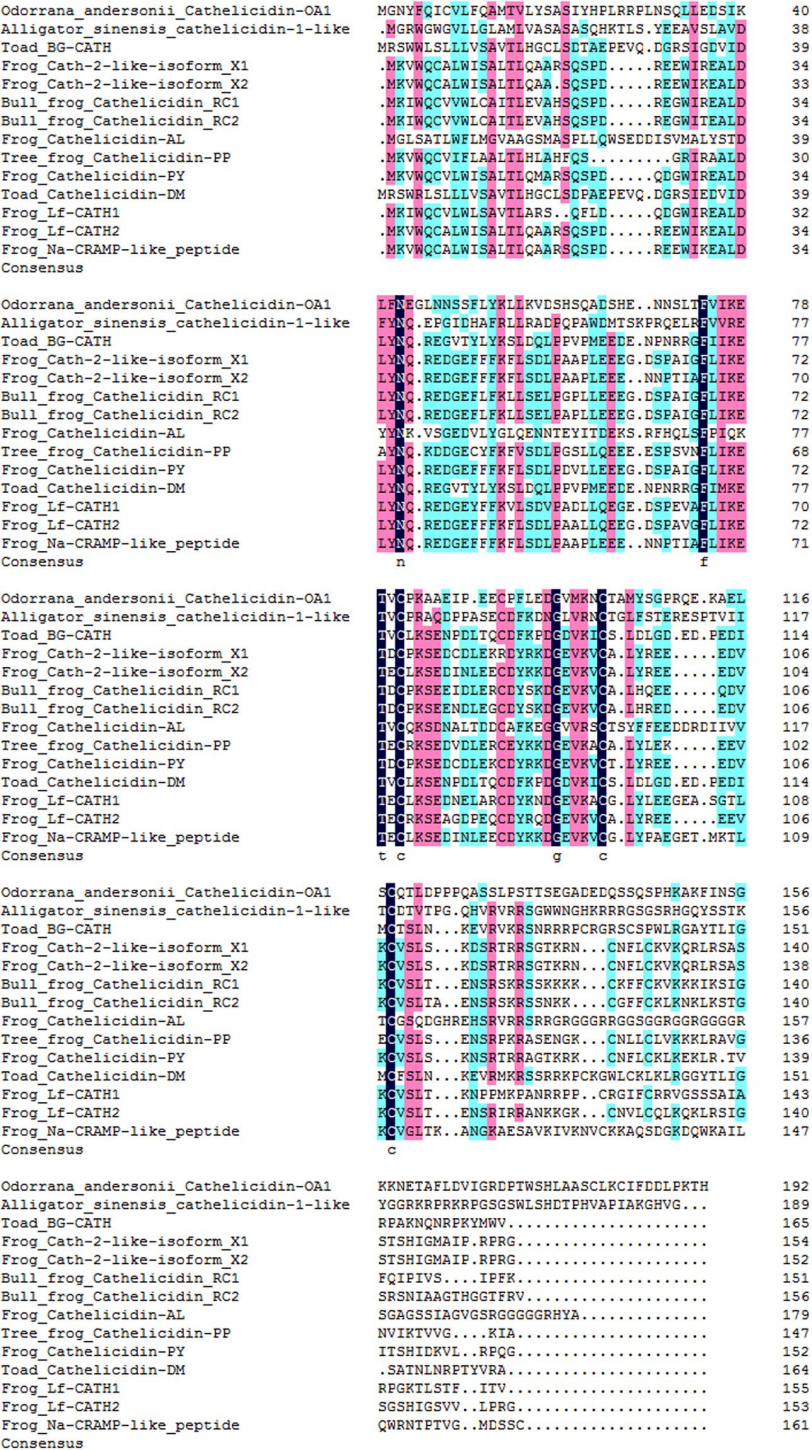
Multiple sequence alignments of cathelicidin-OA1 with several known cathelicidins. Dashes were inserted to optimize alignment.

The sample that demonstrated antioxidant activity (indicated by an arrow in Fig. 1C) was collected and its primary structure was determined. Using an Edman sequencer, the amino acid sequence of this peptide was determined to be ‘IGRDPTWSHLAASCLKCIFDDLPKTHN′ from the N-terminus to C-terminus (Fig. 2A). The cDNA clone encoding the peptide precursor was screened from the skin cDNA library, and well-matched the Edman-determined sequence (GenBank accession number: MF577057). As shown in Fig. 2A, the peptide precursor was composed of 198 amino acid residues. By Blast searching the NCBI database, this peptide showed a sequence similarity of 27% to cathelicidin-1-like from *Alligator sinensis* (Fig. 3), indicating that the precursor was a member of the cathelicidin family. Thus, this novel peptide was named cathelicidin-OA1 (OA: *Odorrana andersonii*). Mass spectrometry revealed that the observed molecular mass (OMM) of cathelicidin-OA1 was 3035.5 Da (Fig. 2B); however, the theoretical molecular mass (TMM), as calculated at http://web.expasy.org/compute_pi/, was 3037.49 Da. This 2 Da difference suggests that cysteine14 and cystine17 might link together to form an intramolecular disulfide bridge. The multiple cathelicidin-OA1 sequence alignments with other several known cathelicidins were shown in Fig. 3. The sequence of mature cathelicidin-OA1 was found to be quite different from the previously identified amphibian cathelicidins, instead of Chinese alligator, *A. sinensis*, indicating that the possible biological functions difference between cathelicidin-OA1 and others.

### Cathelicidin-OA1 showed antioxidant activity

**Figure 4:**
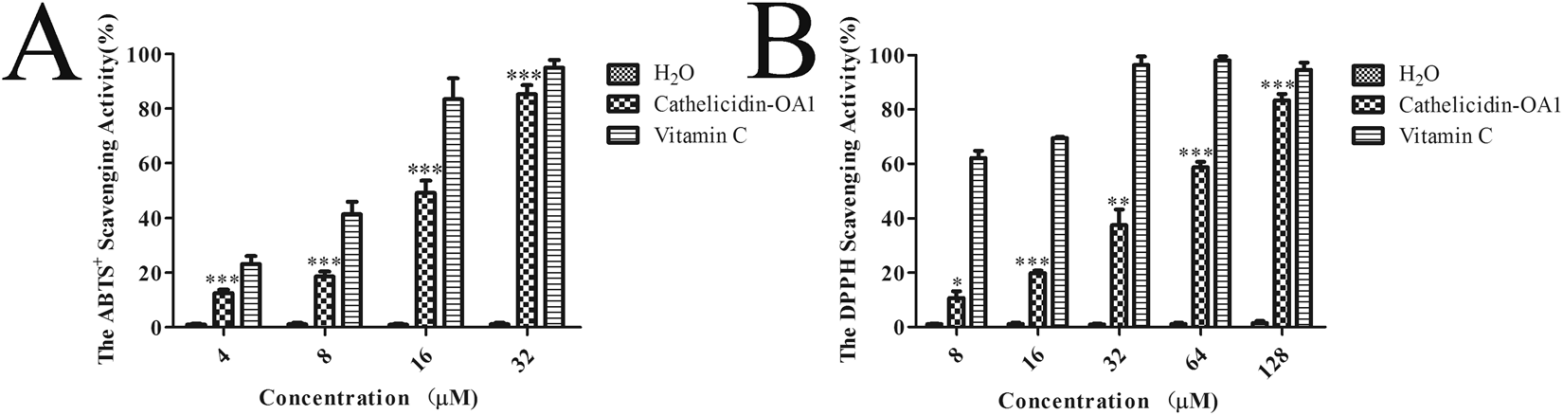
Free radical scavenging activities of cathelicidin-OA1. (**A**) Dose-dependent ABTS^+^ free radical scavenging activity of cathelicidin-OA1. (**B**) Dose-dependent DPPH free radical scavenging activity of cathelicidin-OA1. ‘H_2_O’ indicates negative control and ‘vitamin C’ indicates positive control. *P < 0.05, **P < 0.01, and ***P < 0.0001 indicate significant differences from the negative control (Students *t*-test). Data are mean values of three independent experiments performed in triplicate.

As illustrated in Fig. 4A, cathlicidin-OA1 showed ABTS^+^ free radical scavenging activity in a dose-dependent manner at concentrations of 4, 8, 16, and 32 μM. At a concentration of 4 μM, cathelicidin-OA1 scavenged 12.5% and vitamin C scavenged 23.2% of ABTS^+^. At a concentration of 16 μM, vitamin C scavenged nearly 80% of ABTS^+^, whereas cathelicidin-OA1 scavenged only 49.1% of ABTS^+^. Cathelicidin-OA1 scavenged nearly 90% at a concentration of 32 μM. These results indicated the ABTS^+^ scavenging activity of cathelicidin-OA1. In Fig. 4B, cathelicidin-OA1 also showed DPPH scavenging activity in a dose-dependent manner. At a concentration of 32 μM, vitamin C showed the strongest scavenging activity; however, at a concentration of 128 μM, cathelicidin-OA1 reached nearly 90%. These results demonstrate that cathelicidin-OA1 exhibited antioxidant activity, though its ability was weaker than that of vitamin C. We also tested the antioxidant activity of two other versions of cathelicidin-OA1, liner cathelicidin-OA1 and cathelicidin-OA1 (C14/A), which contained two and one free cysteine residues, respectively. As shown in Fig. S1, compared with naturally-occurred cathelicidin-OA1, both showed an increase in scavenging activities against ABTS^+^ and DPPH.

### Cathelicidin-OA1 showed no antimicrobial activity

We next determined the potency of cathelicidin-OA1 against pathogens. As listed in Table 1, unlike other cathelicidins, cathelicidin-OA1 showed no direct killing effect on gram-positive bacterial strains *Staphylococcus epidermidis*, *S. haemolyticus*, and *Enterococcus faecalis*, gram-negative bacterial strains *Escherichia coli*, *Salmonella paratyphi A*, *Pseudomonas aeruginosa*, *Acinetobacter junii*, *Aeromonas hydrophilia, Vibrio splendidus* and *Streptococcus iniae*, and fungal strains *Candida glabrata* and *C. albicans*, even at a maximum concentration of 1 mM.

**Table 1:** Antimicrobial activity of cathelicidin-OA1.

Microorganisms	Antimicrobial activity	
	AMP	Cathelicidin-OA1
**Gram-positive bacteria**		
*Staphylococcus epidermidis* (ATCC 12228)	+	−
*Staphylococcus haemolyticus* (ATCC 29970)	+	−
**Gram-negative bacteria**		
*Escherichia coli* (ATCC 25922)	+	−
*Salmonella paratyphi A* (ATCC 9150)	+	−
*Pseudomonas aeruginosa* (ATCC 27853)	+	−
*Acinetobacter junii* (ATCC 17908)	+	−
*Aeromonas hydrophilia* (ATCC 49140)	−	−
*Vibrio splendidus* (ATCC 33869)	+	−
*Streptococcus iniae* (ATCC 29177)	+	−
**Fungal strains**		
*Candida glabrata* (ATCC 66032)	+	−
*Candida albicans* (ATCC 14053)	+	−

‘+’: Indicates antimicrobial activity. ‘−’: Indicates no antimicrobial activity.

### Cathelicidin-OA1 accelerated HaCaT cell scratch healing

**Figure 5:**
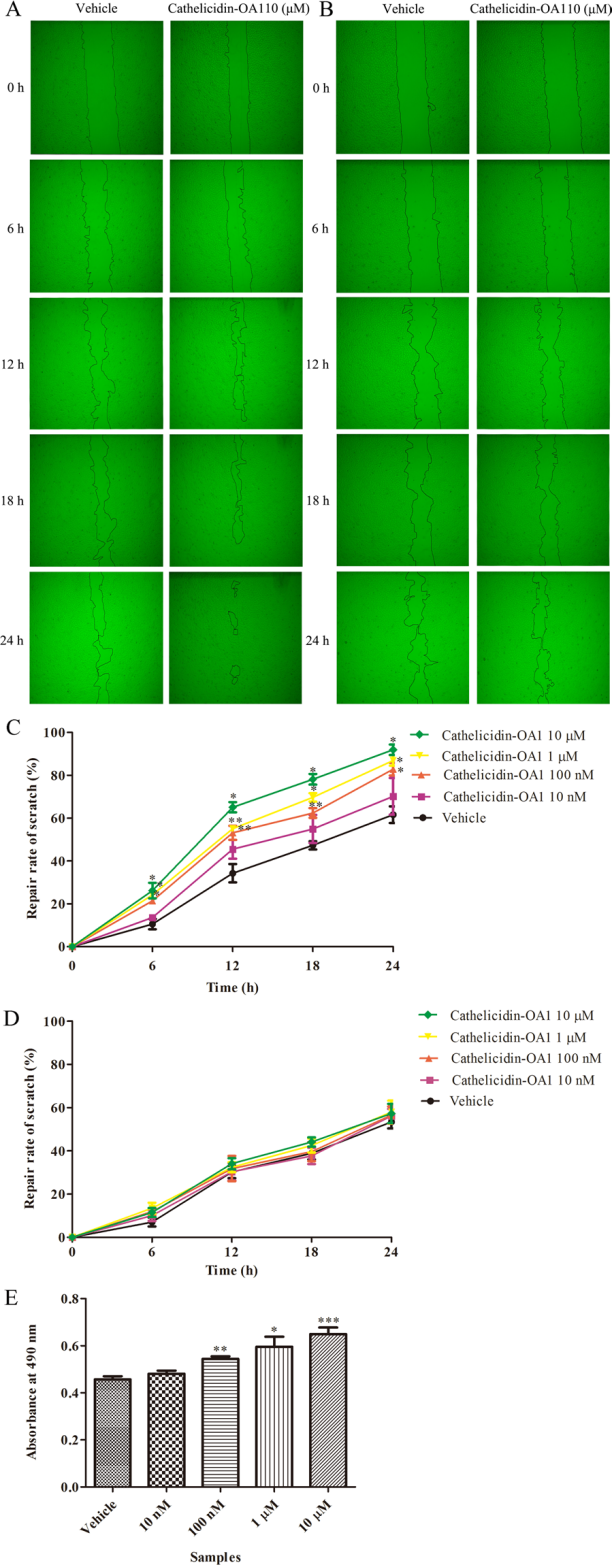
Effect of cathelicidin-OA1 on the repair rate of HaCaT cell wounds (scratches). (**A**) Cathelicidin-OA1 (10 μM) illustrated obvious HaCaT cell wound-healing activity. (**B**) Cathelicidin-OA1 (10 μM) illustrated no obvious HaCaT cell wound-healing activity when proliferation was inhibited by mitomycin C. (**C**) Cathelicidin-OA1 illustrated time- and dose-dependent HaCaT cell wound-healing activity. (**D**) Cathelicidin-OA1 illustrated no time- or dose-dependent HaCaT cell wound-healing activity when proliferation was inhibited by mitomycin C. (**E**) At concentrations of 10 nM to 10 μM, cathelicidin-OA1 showed proliferative effects on HaCaT cells. ‘Vehicle’ is the negative control; *P < 0.05 and **P < 0.01 indicated significantly different from the vehicle (Student’s *t-*test). Data are mean values of three independent experiments performed in triplicate.

Keratinocytes, which are epidermal cells that produce keratin, play vital roles in the healing of cutaneous wounds^15^. Keratinocytes migrate rapidly to wound areas and proliferate to promote re-epithelialization of the wound incision^21^. In the present study, we used an *in vitro* cell scratch assay to investigate the healing effects of cathelicidin-OA1 (at gradient concentrations of 10 nM, 100 nM, 1 μM, and 10 μM) on HaCaT cells from 0 to 24 h at an interval of 6 h. As shown in Fig. 5, cathelicidin-OA1 did not promote wound-healing activity at 10 nM, but did show strong wound-healing activity at 10 μM. Compared with background healing, treatment with cathelicidin-OA1 (10 μM) showed a significant wound-healing rate of 60.1% at 12 h and 91.9% at 24 h, whereas the background healing rate was 34.3% at 12 h and 61.6% at 24 h (Fig. 5A). Cathelicidin-OA1 promoted wound repair in both time- and dose-dependent manners (100 nM–10 μM, Fig. 5A and C). For the scrambled controls, neither cathelicindin-OA1 nor cathelicindin-OA1 (C14/A) showed any effect on the healing of HaCaT cell scratches at the maximum concentrations of 10 μM (Fig. S2). Considering that both the proliferation and migration of HaCaT cells promoted wound healing, we used mitomycin C (10 μg/mL) to inhibit HaCaT cell proliferation, and further determine the effect of cathelicidin-OA1 on HaCaT cell migration. Our results demonstrated that cathelicidin-OA1 did not accelerate wound-healing (Fig. 5B and D) when mitomycin C inhibited HaCaT cell proliferation. Therefore, an MTS assay was employed to investigate the effects of cathelicidin-OA1 on HaCaT cell proliferation. As shown in Fig. 5E, cathelicidin-OA1 promoted the proliferation of HaCaT cells at concentrations of 100 nM, 1 μM, and 10 μM. Based on the cell scratch and MTS assay results, we speculated that cathelicidin-OA1 accelerated keratinocyte wound healing by promoting cell proliferation, rather than affecting cell migration.

### Cathelicidin-OA1 accelerated HSF cell scratch healing

**Figure 6:**
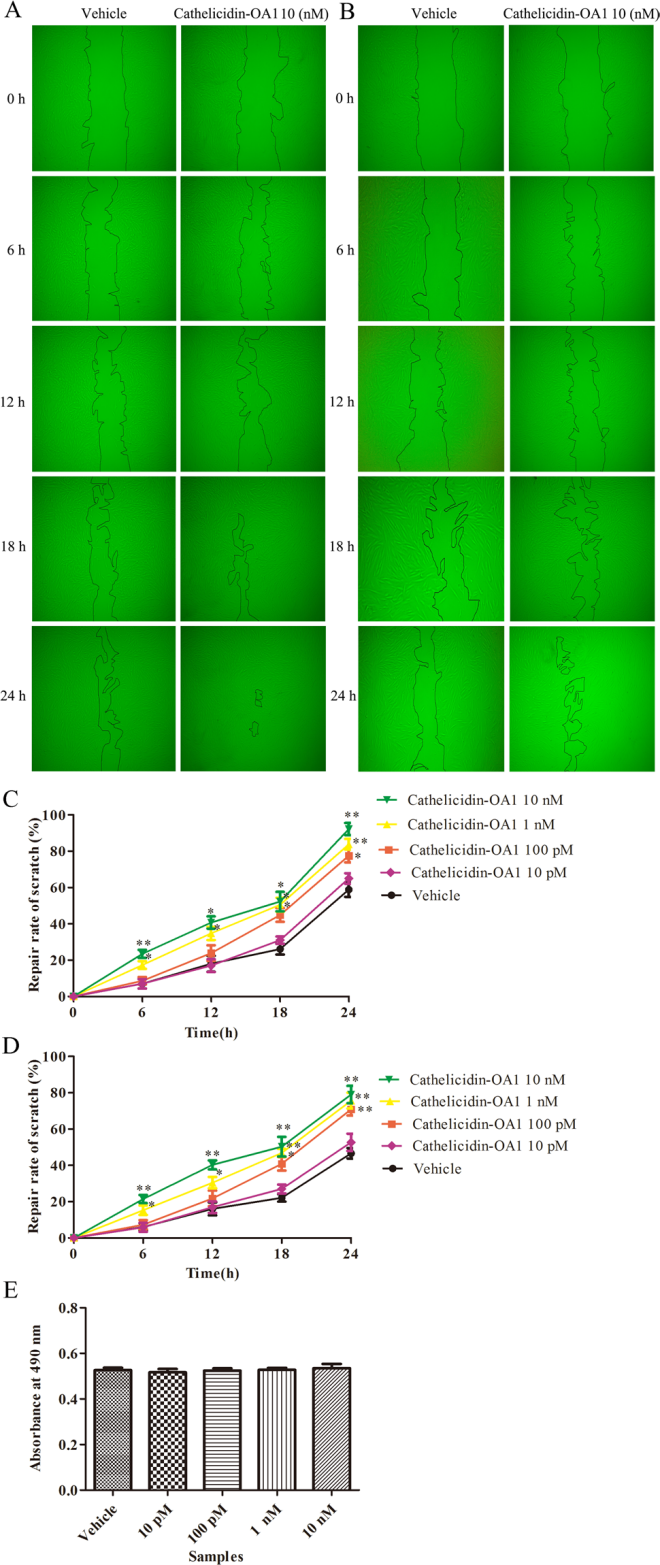
Effect of cathelicidin-OA1 on the repair rate of HSF cell wounds (scratches). (**A**) Cathelicidin-OA1 (10 nM) illustrated obvious HSF cell wound-healing activity. (**B**) Cathelicidin-OA1 (10 nM) illustrated HSF cell wound-healing activity when proliferation was inhibited by mitomycin C. (**C**) Cathelicidin-OA1 illustrated time- and dose-dependent HSF cell wound-healing activity. (**D**) Cathelicidin-OA1 illustrated time- and dose-dependent HSF cell wound-healing activity when proliferation was inhibited by mitomycin C. (**E**) At 10 pM to 10 nM, cathelicidin-OA1 showed no effect on the proliferation of HSF cells. ‘Vehicle’ is the negative control; *P < 0.05 and **P < 0. 01 indicated significantly different from the vehicle (Student’s *t-*test). Data are mean values of three independent experiments performed in triplicate.

In addition to keratinocytes, fibroblasts also play important roles in the healing of cutaneous wounds, in responding to granulation tissue formation and extracellular matrix synthesis^15^. Thus, the effects of cathelicidin-OA1 on the promotion of wound-healing activity in HSF cells were also investigated. At a concentration of 10 nM, cathelicidin-OA1 demonstrated a significant wound-healing promoting rate of 43.7% at 12 h and 92.2 at 24 h, whereas the control showed a healing rate of 18.1% at 12 h and 58.9% at 24 h (Fig. 6A and C). We then tested cathelicidin-OA1 at concentrations of 10 pM, 100 pM and 1 nM, and found that cathelicidin-OA1 accelerated the healing of HSF cell scratches in both a time- and concentration-dependent manner (Fig. 6C). For the scrambled controls, neither cathelicindin-OA1 nor cathelicindin-OA1 (C14/A) showed any effect on the healing of HSF cell scratches at concentrations of 10 nM (Fig. S3). At a concentration of 10 nM, 1 nM, 100 pM and 10 pM, cathelicidin-OA1 also induced wound-healing activity when proliferation was inhibited by mitomycin C (10 μg/mL) (Fig. 6B and D). As displayed in Fig. 6E by an MTS assay, cathelicidin-OA1 didn’t promote HSF cell proliferation. These results indicated that cathelicidin-OA1 promote cell migration, rather than proliferation, to induce the healing of HSF cell scratches.

### Cathelicidin-OA1 accelerated wound healing in a mouse skin wound model

**Figure 7:**
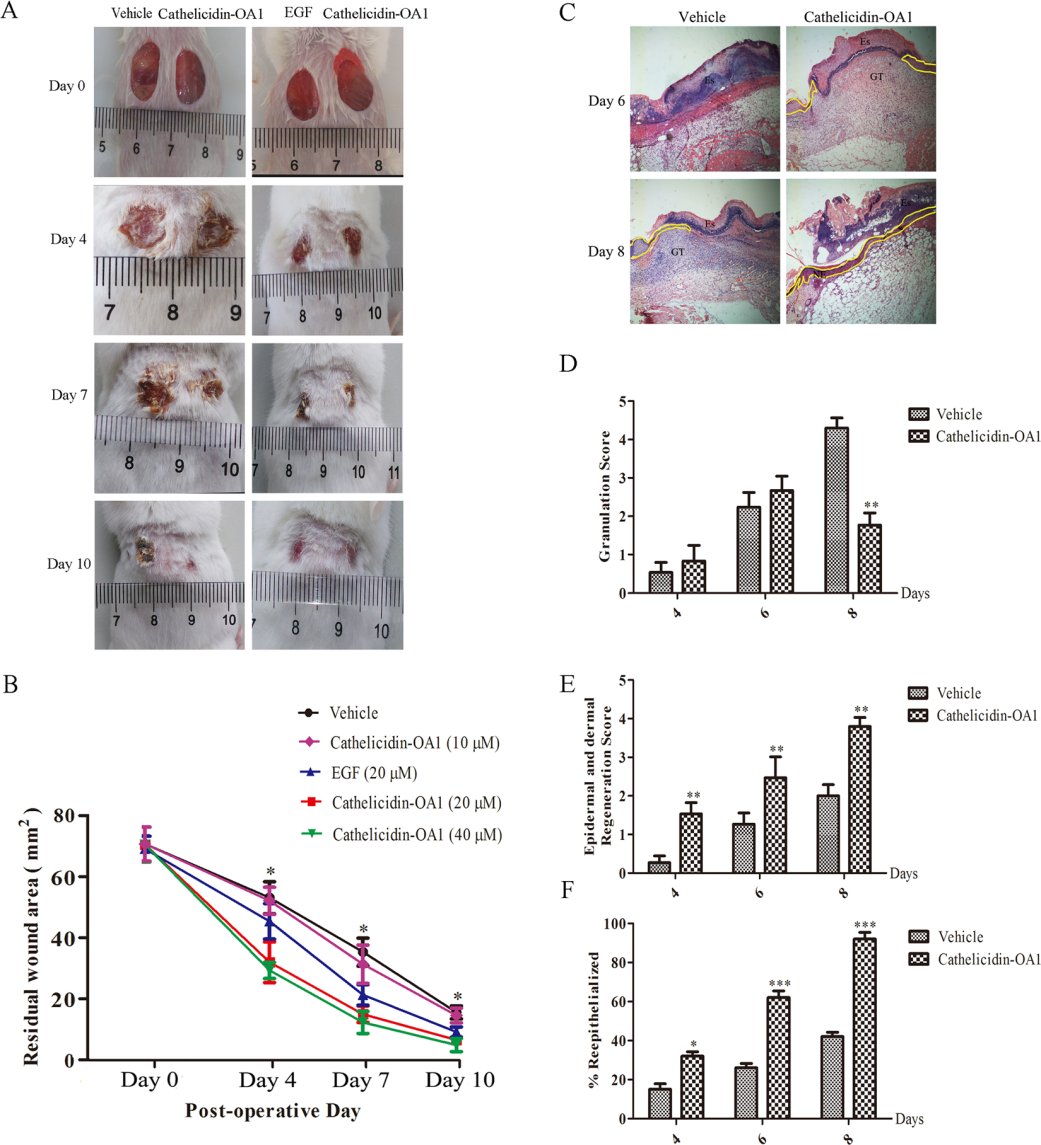
Topical application of cathelicidin-OA1 accelerated healing of full- thickness skin wounds in mice. (**A**) Macroscopic view of wound healing on days 0, 4, 7, and 10. Two 8 × 8 mm wounds were created on the back of each mouse, with wounds treated twice a day with 20 μl of saline (vehicle), 20 μM cathelicidin-OA1, or 20 μM EGF and 20 μM cathelicidin-OA1, respectively. Images of representative mice were taken on days 0, 4, 7, and 10 post-operation. (**B**) Cathelicidin-OA1 illustrated time- and dose-dependent wound-healing activity in the mouse model. Wound closure was assessed by morphometrical analysis of wound areas (Image J, NIH). Wound residual area was determined (n = 10) from three independent experiments. *P < 0.05 indicates the group treated with cathelicidin-OA1 (20 μM and 40 μM) was significantly different from the negative control (Students *t-*test). (**C**) Histopathological examination of vehicle and cathelicidin-OA1-treated (20 μM) healing skins (post-operative days 5 and 9) stained with H&E. Neo-epithelium marked by yellow dotted line was much longer at the same magnitude of enlargement in cathelicindin-OA1-treated mice than in vehicle controls (NE: neo-epithelium; GT: granulation tissue; Es: eschar). (**D**,**E**,**F**) Epidermal thickness in mice; histological scores of granulation thickness and epidermal and dermal regeneration. All bars represent means ± SD from three independent experiments and six different sections for each experiment. *P < 0.05, **P < 0.01 and ***P < 0.0001 (cathelicidin-OA1 treatment vs control).

As cathelicidin-OA1 significantly promoted the healing of cell scratches *in vitro*, its wound-healing activity was evaluated *in vivo* using a full-thickness skin excisional wound repair mouse model. Cathelicidin-OA1 (10, 20, and 40 μM, 20 μl), epidermal growth factor (positive control, EGF, 20 μM, 20 μl), and saline (negative control, 20 μl) were applied to wounds twice daily, respectively. The application of 20 μl of cathelicidin-OA1 (20 μM and 40 μM, but not 10 μM) on mice significantly promoted wound healing compared with the vehicle control and showed similar wound-healing promoting activity as that of EGF (Fig. 7A). At 20 μM, residual wounds were 32, 15.02, and 6.63% of the original size in cathelicidin-OA1-treated mice compared with 53.5, 35.3, and 15.6% in vehicle-treated mice on postoperative days 4, 7, and 10, respectively. In addition, residual wounds were 36.4, 15.7, and 0% of the original size in cathelicidin-OA1-treated mice, compared with 33.9, 12.8, and 0% in EGF-treated mice on postoperative days 4, 7, and 10, respectively. During the wound healing process, there were no adverse effects on mouse body weight, general health, or behavior following cathelicidin-OA1 treatment (data not shown). In summary, cathelicidin-OA1 significantly accelerated the healing of wounds *in vivo* compared with the saline group and showed similar potency to that of EGF.

In a parallel experiment, mice were euthanized at postoperative days 4, 6, and 8, and wound specimens, including a 5-mm margin of non-wounded skin from the wound site, were removed from cathelicidin-OA1-treated mice and untreated controls after sacrifice, and then fixed in 10% formalin. We cut 5-μm thick sections and stained them with H&E for histological analysis. The cathelicidin-OA1-treated mice showed obvious accelerated regeneration of neo-epidermal tissue (neo-epithelial tongue) and restoration of dermis (granulation tissue formation) in the wound. As illustrated in Figs 7C–F, two main differences between cathelicidin-OA1 and vehicle treatment were observed: re-epithelialization was accelerated and granulation tissue contraction was stronger in cathelicidin-OA1-treated wounds compared with vehicle-treated wounds.

### Effect of cathelicidin-OA1 on TNF-α and TGF-β1 secretions

**Figure 8:**
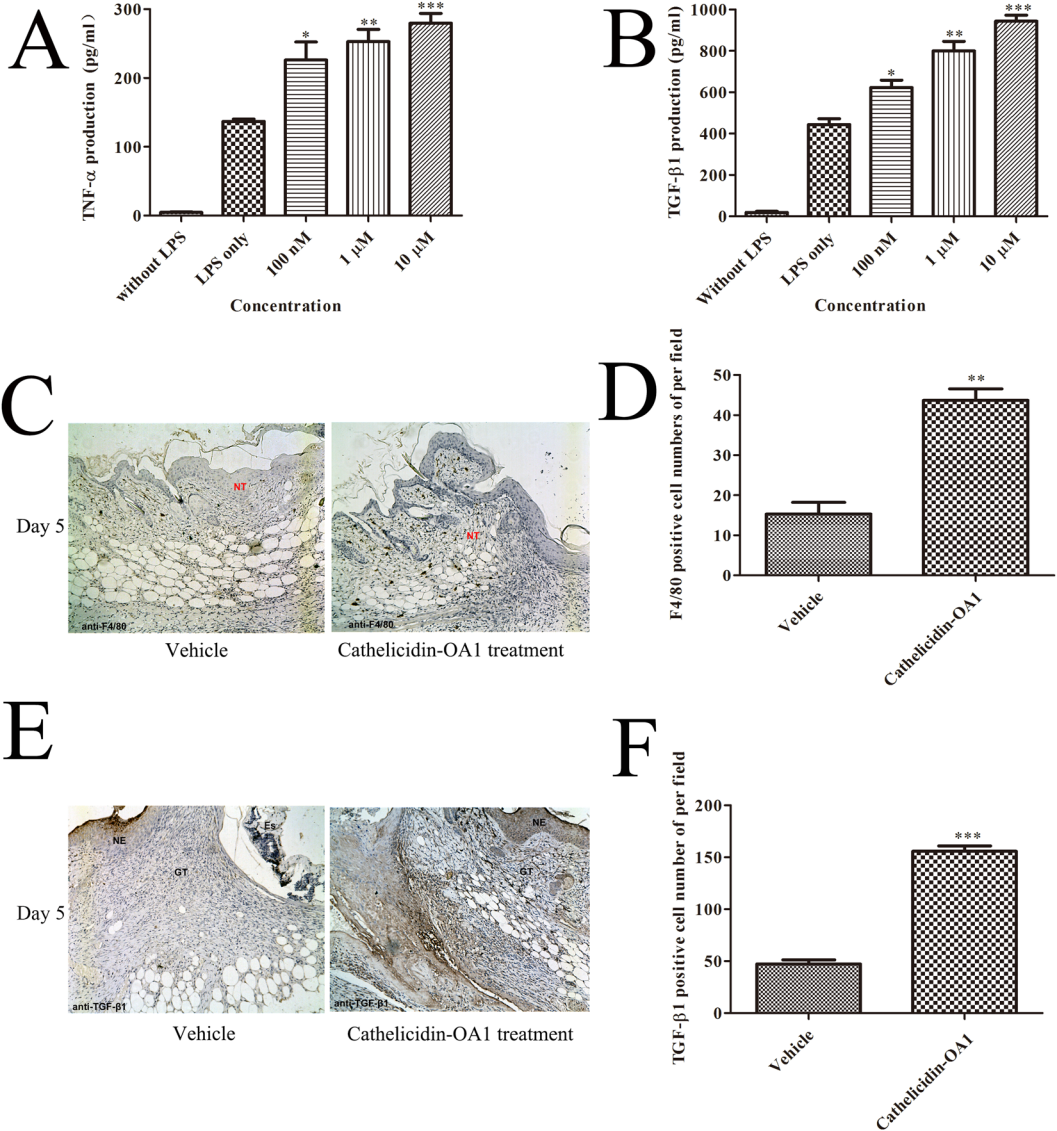
Cathelicidin-OA1 promoted the secretion of TNF-α and TGF-β, macrophages recruitment, TGF-β expression. (**A**,**B**) THP-1 cells were stimulated by LPS to secrete TNF-α and TGF-β1. Incubation with cathelicidin-OA1 at different concentrations resulted in the dose-dependent increase in TNF-α and TGF-β1 secretion. *P < 0.05, **P < 0.01, and ***P < 0.0001 indicate significantly different from the negative control (Student’s *t*-test). Data are mean values of three independent experiments performed in triplicate. (**C**,**E**) Immunohistochemical results for anti-F4/80 and TGF-β1. (**D**,**F**) F4/80 or TGF-β1 positive cell numbers per high power field were significantly different between cathelicidin-OA1 treatment and the control. **P < 0.01 and ***P < 0.0001. Data are mean values of three independent experiments performed in triplicate and six different fields for each section (×100).

TNF-α, a pro-inflammatory cytokine, can be timely upregulated in the early phase of inflammation during wound healing, which is responsible for recruiting inflammatory cells to wound site and exerting predominant chemotactic functions. As for TGF-β1, an extremely important transforming growth factor during the whole processes of wound healing, can orchestrate the complex processes of cellular proliferation, migration, and functional tissue regeneration within wound^22^. To investigate the possible molecular mechanism of cathelicidin-OA1 on wound healing, we tested the effects of cathelicidin-OA1 on TNF-α and TGF-β1 secretions induced by LPS in THP-1 cells. As illustrated in Fig. 8A and B, although LPS alone significantly induced TNF-α and TGF-β1 secretions. After the incubation of cathelicidin-OA1 at concentrations of 100 nM, 1 μM, and 10 μM for 12 h, the TNF-α concentration in the supernatant increased in a dose-dependent manner from 226.3 to 252.8 and 280.1 pg/mL, respectively. In addition, TGF-β1 secretion also increased in a dose-dependent manner in the cathelicidin-OA1-treated cells from 623.3 to 800 and 943.7 pg/mL, respectively (Fig. 8B).

Macrophages are involved in each phase of wound healing, including host defense, inflammation promotion and resolution, cell proliferation, and tissue restoration following injury^23^. Immunohistochemical analysis revealed that many macrophages were recruited to the unwounded skin margin near the neo-epidermis and neo-dermis in cathelicidin-OA1-treated skin wounds (Fig. 8C). Compared with the ~18 F4/80 (a specific macrophage marker)-positive cells per field in vehicle-treated skin wounds, we detected ~50 F4/80-positive cells per field in the cathelicidin-OA1-treated skin wounds (Fig. 8D). Immunohistochemical analysis also showed that TGF-β1-positive stained cells were much more intensive in the cathelicidin-OA1-treated group compared with the vehicle controls (Fig. 8E). We detected ~45 and ~150 TGF-β1-positive stained cells per field in the vehicle- and cathelicidin-OA1-treated groups, respectively (Fig. 8F).

Taken together, these findings implied that cathelicidin-OA1 induced TNF-α secretion in the early inflammatory phase, resulting in an increase in macrophage recruitment. With more macrophages recruited to the wound site, additional TGF-β1 was secreted, which facilitated wound healing.

### Hemolysis and toxicity of cathelicidin-OA1

Considering the possible clinical use of cathelicidin-OA1 as a wound-healing promoting candidate, we used fresh human erythrocytes to test the hemolytic activities of cathelicidin-OA1. Cathelicidin-OA1 displayed negligible hemolytic activity (<10%) on human erythrocytes, even at concentration of 100 μM (Table 2). We also investigated an *in vivo* mouse model with a single intraperitoneal injection of cathelicidin-OA1 at concentrations of 5, 10, 20, and 40 μmol/kg, and found no lethal effects, including abnormal behaviors such as tremor, retardation, irritability, piloerection, and stiffness of tail, after two weeks (Table 3). These results suggested the potential safety of cathelicidin-OA1 for therapeutic application.

**Table 2:** Hemolytic activity of cathelicidin-OA1 in human erythrocytes.

Group	Hemolytic ratio
Triton X-100	100%
1 nM cathelicidin-OA1	5.4% ± 0.14%
10 nM cathelicidin-OA1	4.5% ± 0.61%
100 nM cathelicidin-OA1	5.4% ± 0.81%
1 μM cathelicidin-OA1	6.9% ± 0.57%
10 μM cathelicidin-OA1	5.0% ± 0.86%
100 μM cathelicidin-OA1	7.7% ± 0.11%

Values are means ± SEM, n = 6.

**Table 3:** Mouse mortality at different inoculation doses of Cathelicidin-OA1.

Group	Dosage (μmol/kg)	Number of mice		Mortality rate (%)
		male	female	
Negative control (saline injection)	40	5	5	0
Experimental group				
Group 1	5	5	5	0
Group 2	10	5	5	0
Group 3	20	5	5	0
Group 4	40	5	5	0

## Discussion

Wound healing is a highly complex process, with many critical factors influencing normal wound recovery and impaired wound healing caused severe consequences, even death^24^. With the increase in chronic wounds due to accidents, aging populations, and incidences of diseases such as diabetes, wound healing continues to be a considerable challenge in clinical treatment^14^. Although several drugs reportedly promote wound healing, including vascular endothelial growth factor (VEGF) and erythropoietin (EPO), they have yet not to achieve ideal effects^25^. Thus, identifying novel molecules for the treatment of wounds is still of great necessity. Compared with traditional drugs, peptides exhibit many advantages such as high activity, specificity, and stability, and have thus provoked considerable scientific attention worldwide^14^.

Amphibian skin secretions display considerable bioactivity, including antioxidant, antimicrobial and wound-healing activities, and are therefore worthy of development in novel medical treatment^26,27^. Odorous frog skins secrete various biologically active peptides and have a high number of AMPs. However, AMPs only account for a small portion of skin peptides, with many unidentified peptides awaiting development^17^. Considering that odorous frogs live in high-elevation environments where their skins are exposed to elevated ultraviolet (UV) radiation, they likely possess a specific and highly effective antioxidant system. The initial goal of the current research was to identify novel antioxidant peptides from *O. andersonii*. We tested the ABTS^+^ free radical scavenging activities of fractions obtained from the skin secretions of *O. andersonii* via gel filtration chromatography. Fractions that showed ABTS^+^ free radical scavenging were further purified using RP-HPLC procedures (Fig. 1). Thus, a peptide with ABTS^+^ free radical scavenging activity was obtained from *O. andersonii* secretions. Through Edman sequencing and cDNA cloning (Fig. 2A), the complete amino acid sequence of this peptide was ‘IGRDPTWSHLAASCLKCIFDDLPKTHN′, with an intramolecular disulfide bridge indicated by mass spectrometry (Fig. 2B). We performed Blastp searching of the NCBI database and determined that this peptide belonged to the cathelicidin family, and was therefore named cathelicidin-OA1. By sequence alignments, mature cathelicidin-OA1 showed no obvious sequence similarities with other amphibian cathelicidins, but did with reptilian cathelicidins- *A. sinensis* cathelicidin-1-like peptide (Fig. 3). This indicated that cathelicidin-OA1 might facilitate different biological functions.

Cathelicidin-OA1 showed obvious scavenging activity against ABTS^+^ and DPPH free radicals (Fig. 4). Compared with other peptides from odorous frogs, such as adersonin-AOP1^34^, the antioxidant activity of cathelicidin-OA1 was slightly weaker. However, when the intramolecular disulfide bridge of cathelicidin-OA1 was disrupted, the antioxidant activities of linear cathelicidin-OA1 (containing two free cysteine residues) and cathelicidin-OA1 (C14/A) were significantly enhanced (Fig. S1). Together with our previous research, we concluded that free cysteines do not ensure but do enhance the antioxidant activities of a specific peptide^27^. Except for tylotoin, amphibian cathelicidins often exhibit direct microbe-killing effects^15^. In this report, we used three gram-positive bacterial strains, seven gram-negative bacterial strains and two fungal strains to test the antimicrobial activities of cathelicidin-OA1. Results showed that cathelicidin-OA1 did not exhibit antimicrobial activity against any of these strains. These results demonstrated that cathelicidin-OA1 does not possess antimicrobial activity, and might therefore serve another function.

*Odorrana andersonii* lives in relatively harsh environments, which can increase the incidence of skin damage^14^. Thus, given the high UV-irradiative plateau habitats of *O. andersonii*, cathelicidin-OA1 may facilitate frog survival in severe environments. As previous research indicates that cathelicidins can facilitate wound healing^15^, we examined the effect of cathelicidin-OA1 on wound repair, and found it to exhibit significant wound-healing activity. Cathelicidin-OA1 accelerated the healing of HaCaT and HSF cell scratches in both time- and dose-dependent manners (Figs 5 and 6), though at different concentrations. Different working mechanisms might be involved in the closure of different cell injuries. We demonstrated that cathelicidin-OA1 promoted HaCaT cell proliferation and HSF cell migration, the mechanisms of which are currently being studied. At present, we are investigating whether cathelicidin-OA1 can promote integrin receptor expression in HSF cell membranes, which may help clarify the promotion of cell migration by cathelicidin-OA1. Simultaneously, we are also exploring the correlative cell signal pathways, which may help elucidate how cathelicidin-OA1 influences HaCaT cell proliferation. As scrambled controls, linear cathelicidin-OA1 and cathelicidin-OA1 (C14/A) showed no effects on the healing of HaCaT and HSF cell scratches (Figs S2 and S3), indicating that the effect of cathelicidin-OA1 is sequence-specific, composition-dependent, or conformation-specific. In conclusion, the mechanism by which cathelicidin-OA1 accelerates cellular wound healing activity is complicated and needs to be further investigated.

Promisingly, cathelicidin-OA1 demonstrated strong wound-healing promotion in the full-thickness skin-wound mouse model. Gradient concentrations of cathelicidin-OA1 (10, 20, and 40 μM) were separately tested on full-thickness skin wounds. Treatment with the minimum concentration of cathelicidin-OA1 (20 μM and 40 μM) strongly accelerated skin wound healing in mice. Histological analysis indicated that cathelicidin-OA1 accelerated re-epithelialization capability and promoted granulation tissue formation in the early stage of skin wound healing, but granulation tissue contraction in the later period (Fig. 7). Cathelicidin-OA1 showed strong wound-healing activity compared with that of the vehicle group, and similar wound healing effects compared with EGF at the same concentration *in vivo*.

The inflammation stage plays a key role in whole stages of wound repair^28^. During inflammation phase, inflammatory cell infiltration, especially that of macrophages, can lead to the production of various proinflammatory cytokines and growth factors^15,28^. In the early stage of inflammation, proinflammatory cytokines can recruit additional inflammatory cells, ensuring that growth factors released by macrophages can regulate the migration and proliferation of repair cells, like keratinocytes and fibroblasts^29^. Therefore, proinflammatory cytokines, like TNF-α, IL-6, and IL-1, can effectively promote transition from inflammation to cell proliferation^15,24^. In our study, cathelicidin-OA1 increased TNF-α release over the short-term, which may have contributed to recruit macrophages to wound site to accelerate the wound repair. As shown in Fig. 8, compared with the negative control, immunohistochemical staining demonstrated much more macrophages were recruited to the impaired skin margin closed to neo-epidermis and neo-dermis in cathelicilin-OA1-treated skin wound. These results suggest that cathelicidin-OA1 markedly accelerated TNF-α release and recruited macrophages to the unwounded skin margin near the neo-epidermis and granulation tissue, which, in turn, enhanced wound healing via TGF-β1 secretion (Fig. 8E). In previous study, the wound-healing peptide AH-90 also induced TNF-α secretion during inflammation and played an important role in initiating healing and wound re-epithelialization^21^. It was manifested that peptide cathelicidin-OA1 strongly effected on macrophages recruitment, TNF-α releasing, TGF-β expression, resulted in its potential wound-healing ability.

## Conclusions

In this research, a novel peptide was identified from skin secretions of the odorous frog *O. andersonii*. This cathelicidin-OA1 peptide (IGRDPTWSHLAASCLKCIFDDLPKTHN) showed no antimicrobial activity, hemolytic activity, or acute toxicity, but did exhibit antioxidant activity. Of note, significant wound healing potency was demonstrated *in vivo* and *in vitro*. Our results provide a novel peptide candidate for the development of wound-healing promoting agents.

## Materials and Methods

### Sample collection and animal care

Adult *O. andersonii* specimens (n = 30) were collected from Baoshan City in Yunnan Province, China, and transferred to the laboratory. Before the experiments commenced, the frogs were housed together in a 50 cm × 60 cm box for several days and provided with mealworms *ad libitum*. After one week, skin secretions were collected. The frogs were put in H_2_O containing 0.01% NaCl and then stimulated using an electronic massager with an alternating current (6 V) for 6 s. Skin secretions were then collected by washing the frog bodies with 25 mM Tis-HCl buffer (pH 7.8). The collected secretions were centrifuged at 4000 g for 15 min at 4 °C, with the resulting supernatants collected and lyophilized. The samples were stored at −80 °C until further analysis.

All study protocols and procedures were approved by the Ethics Committee of Kunming Medical University, and were conducted in accordance with the guidelines for Animal Care and Use at Kunming Medical University.

### Purification procedure

Purification was based on previous peptide purification procedures^17^. Briefly, the lyophilized skin secretions were re-dissolved (500 µl, OD_280_ = 30) in water and placed into a Sephadex G-75 (1.5 × 31 cm, superfine, GE Healthcare, Sweden) gel filtration column equilibrated with 25 mM Tris-HCl buffer (pH 7.8) containing 0.1 M NaCl as the elution buffer at a flow rate of 0.1 ml/min. An automatic fraction collector (BSA-30A, Huxi Company, Shanghai, China) accumulated fractions every 10 min, with absorbance monitored at 280 nm. The antioxidant activities of the fractions were then tested against ABTS^+^ free radicals. Fractions that exhibited antioxidant activity were combined and placed into a C18 RP-HPLC column (Hypersil BDS C18, 4.0 × 300 mm, Elite, China) pre-equilibrated with 0.1% (v/v) trifluoroacetic acid (TFA) in water. Elution was performed using a linear gradient (0–60% acetonitrile in 60 min) of 0.1% (v/v) TFA in acetonitrile at a flow rate of 1 ml/min and monitored at 220 nm. Peaks with antioxidant activities against ABTS^+^ free radicals were collected to track the peptides.

### Determination of peptide primary structure

The monoisotopic molecular mass and purity of native cathelicidin-OA1 was determined using an AXIMA-CFRTM plus MALDI-TOF mass spectrometer (Shimadzu/Kratos, Manchester, UK) in linear mode with α-cyano-4-hydrorycinnamic acid as the matrix. All procedures were carried out per the manufacturer’s standard protocols, and data were analyzed using the provided software package. To obtain the complete amino acid sequence, the peptide was subjected to Edman degradation on a PPSQ-31A protein sequencer (Shimadzu, Japan) according to the manufacturer’s standard GFD protocols, and recognition of cysteine residues was conducted according to the procedures provided by the manufacturer.

### Cloning of cDNA encoding cathelicidin-OA1

An *O. andersonii* skin cDNA library was successfully constructed in our previous research^17^, with the cDNA encoding mature peptide here screened from this library. Briefly, the 5′ PCR primer (5′-CCAAA(G/C)ATGTTCACC(T/A)TGAAGAAA-3′) and 3′ PCR primer (5′-ATTCTAGAGGCCGAGGCGGCCGACATG-3′) were commercially synthesized by the BGI Company (China). PrimeSTAR^®^ HS DNA polymerase (TaKaRa Biotechnology Co., Ltd., Dalian, China) was selected for polymerase chain reaction (PCR) under the following conditions: 2 min at 94 °C and then 25 cycles of 10 s at 92 °C, 30 s at 50 °C, and 40 s at 72 °C. The PCR products were recovered using a DNA gel extraction kit (Bioteke, China) and ligated into a pMD19-T vector (TaKaRa Biotechnology Co., Ltd., Dalian, China). The PCR products were finally cloned into *E. coli* DH5α and independent clones were chosen to carry out DNA sequencing on an Applied Biosystems DNA sequencer (ABI 3730XL, Foster City, CA, USA).

### Peptide synthesis

The mature cathelicidin-OA1 peptide (IGRDPTWSHLAASCLKCIFDDLPKTHN), with an intramolecular disulfide bridge, was commercially synthesized by GL Biochem Ltd. (Shanghai, China) and Wuhan Bioyeargene Biotechnology Co., Ltd. (Wuhan, China). The synthesized cathelicidin-OA1 was analyzed using a mass spectrometer. It was co-eluted with natural cathelicidin-OA1 and confirmed to be identical with the native cathelicidin-OA1. The biological activities of the synthesized cathelicidin-OA1 were then tested. In addition, two scrambled peptides, linear cathelicidin-OA1 and cathelicindin-OA1 (C14/A) (IGRDPTWSHLAASaLKCIFDDLPKTHN), were also synthesized by Wuhan Bioyeargene Biotechnology Co., Ltd. (Wuhan, China).

### Antioxidant activity

A 2, 2-azino-bis (3-ethylbenzothiazoline-6-sulfonic acid) (ABTS) scavenging test was performed as described previously^27^, with some modification. Briefly, a stock solution of ABTS radical was prepared by incubating 2.8 mM potassium persulfate (Sigma-Aldrich, St Louis, MO, USA) with 7 mM ABTS in water for at least 6 h in the dark, after which it was used immediately. The stock solution was diluted 50-fold with ddH_2_O. Samples dissolved in water were added to the diluted stock solution, and the same volume of solvent was used as the negative control. Vitamin C dissolved in H_2_O was used as a positive control. The reaction was kept from light for 30 min. A decrease in absorbance at 415 nm indicated antioxidant activity of the samples. The rate of free radical scavenging (%) was calculated by (A_blank_ − A_sample_) × 100/A_blank_.

A 2, 2-diphenyl-1-picrythydrazyl (DPPH) free radical scavenging test using a stable DPPH radical (Sigma-Aldrich, St Louis, MO, USA) was performed as described previously^30^, with some modification. The assay mixture contained 190 μl of 5 × 10^−5^ M DPPH radical dissolved in ethanol and 10 μl of sample solution. The mixture was then incubated in a 96-well microtiter plate at room temperature for 30 min. Afterwards, the absorbance was read against a blank at 517 nm. Vitamin C was used as the positive control and ddH_2_O as the negative control. The DPPH free radical scavenging activity (%) was calculated by (A_blank_ − A_sample_) × 100/A_blank_.

### Antimicrobial activity assay

The antimicrobial activity of cathelicidin-OA1 was assayed based on our previous research^31^. Briefly, gram-positive bacterial strains *Staphylococcus epidermidis* (ATCC 12228), *Staphylococcus haemolyticus* (ATCC 29970), and *Enterococcus faecalis* (ATCC 29212), gram-negative bacterial strains *Escherichia coli* (ATCC 25922), *Salmonella paratyphi A* (ATCC 9150), *Pseudomonas aeruginosa* (ATCC 27853), *Acinetobacter junii* (ATCC 17908), *Aeromonas hydrophilia* (ATCC 49140, *Vibrio splendidus* (ATCC 33869), and *Streptococcus iniae* (ATCC 29177), and fungal strains *Candida glabrata* (ATCC 66032) and *Candida albicans* (ATCC 14053) were obtained from the Kunming Medical University. The microbes were grown in Luria Bertani (LB) broth to OD_600_ = 0.8. A 10 μl aliquot of each bacterium was collected and added to 10 ml of fresh LB broth with 1% Type I agar (Sigma-Aldrich, St Louis, MO, USA), then poured over a 90-mm Petri dish. When the agar hardened, a small hole was made and a 7 μl aliquot of cathelicidin-OA1 (1 mM) was added to the hole, followed by incubation for 16–18 h at 37 °C. If the sample contained antimicrobial activity, a clear zone appeared on the surface of the agar, representing inhibition of bacterial growth.

### Cellular wound-healing activity assay

Cellular wound-healing activity was determined according to earlier research^28^, with some modification. Briefly, immortalized HaCaT and HSF cells were grown in DMEM/F12 medium (BI, Israel) with 10% fetal bovine serum (FBS) and penicillin (100 U/mL)-streptomycin (100 U/mL) in a humidified atmosphere of 5% CO_2_ at 37 °C. The HaCaT and HSF cells were seeded in 24-well tissue culture plates (2.5 × 10^5^ cells/well) for 12–14 h to achieve monolayer formation. A yellow 200 μl pipette tip (Axygen, USA) was then used to make a mechanical scratch wound, after which the plates were twice washed with PBS to remove any floating cells. Serum-free DMEM/F12 medium (500 μl) containing different concentrations of cathelicidin-OA1 (10 nM, 100 nM, 1 μM, 10 μM for HaCaT and 100 pM, 1 nM, 10 nM for HSF) was then added to each well with or without mitomycin C (10 μg/mL, Sigma-Aldrich, St Louis, MO, USA).

Wound healing images of the monolayers were obtained using a Primovert microscope (Zeiss, Germany) at time intervals of 0, 6, 12, 18, and 24 h. We measured the percentage of the gap relative to the total area of the cell-free region to evaluate cell migration activity, with this percentage considered the repair rate of scarification, using Image J software (National Institutes of Health, Bethesda, MD, USA). For each plate, six randomly selected pictures were acquired. All experiments were independently conducted in triplicate.

### HaCaT and HSF cell proliferation assays

Cells were cultured in DMEM/F12 medium with 10% FBS serum, 100 U/mL of streptomycin, and 100 U/mL of penicillin in a humidified atmosphere of 5% CO_2_ at 37 °C. The cells (5,000 HaCaT cells/well and 10,000 HSF cells/well, 90 μl, respectively) were then added to 96-well plates and incubated for 2–4 h in DMEM/F12 medium-serum to allow the cells to adhere to the well walls. Subsequently, 10 μl of cathelicidin-OA1 was added to each well at various concentrations (10 nM, 100 nM, 1 μM, and 10 μM for HaCaT cells, and 100 pM, 1 nM, and 10 nM for HSF cells), respectively, followed by incubation for 24 h. The same volume of DMEM (serum free) was used as a blank control. After incubation, the effect on HaCaT and HSF cell proliferation was tested using the CellTiter 96® AQueous One Solution Assay (Promega, Madison, WI, USA) according to the manufacturer’s instructions^17^.

### Animal wound-healing assay

Adult male mice (22–25 g) from the same generation were obtained from the Experimental Animal Central of Kunming Medical University for use in the wound-healing model. Mice were housed in individual cages at room temperature and provided with free access to water and laboratory food. Mice were given 7 d to adapt to the laboratory environment before the start of the experiments. The mice were randomly divided into two groups, with 10 mice used per time point in each group for the study, and triplicate experiments conducted independently. The mice were anesthetized using 100 ml of 1% pentobarbital sodium solution (0.1 ml/20 g body weight) by intraperitoneal (i.p.) injection. The dorsal hair of the animals was shaved, with the area then sterilized with 75% alcohol to avoid wound infection. Lastly, two 8 × 8 mm full-thickness skin wounds were surgically made on the back of each animal. At the end of the surgical procedure, the mice were placed in cages near a heating apparatus until they recovered from the anesthesia. The right-sided wounds were treated with 20 μl (20 μM) of cathelicidin-OA1, with the same volume of saline used to treat the wound on the left. Wounds were treated twice daily and were imaged at time intervals every 3 d.

### Wound healing measurement

We used a D3000 digital camera (Nikon, Japan) to record the closure of wounds and Image J software (NIH, USA) to estimate the wound areas (percentage of residual wound area to original wound area). Residual Wound Area (%) = [R (0, 4, 7, 10)/R(0)] × 100%, where R(0) and R(4, 7, 10) denote the remaining wound area at the same day of operation and postoperative days 4, 7, and 10, respectively. Wound-repair curves were constructed using GraphPad Prism software (v.5).

### Tissue preparation and histological analysis

Mouse skin tissues were isolated from the central wound area on different days after wounding, and underwent light microscopy analysis. Tissue samples were fixed in 10% buffered formalin overnight, dehydrated through a graded series of ethanol (70% for 12 h, 80% for 12 h, 90% for 12 h, 95% for 3 h, and100% for 3 h), cleared in xylene for 1 h, and then embedded in paraffin (54–56 °C for 1 h and 58–60 °C for 3 h). Thick sections (5 μm) were cut on positively charged slides, deparaffinized, rehydrated, and stained with hematoxylin and eosin (H&E) for histological analysis. IPLab imaging software (BD Biosciences, Bedford, MA, USA) was used to measure changes in wound areas. In H&E stained sections, wound widths and distances of the neo-epithelium were measured, and the percentage of re-epithelialization (distance covered by neo-epithelium)/(distance between wound bed) × 100 (*n* *=* 6/group) was calculated.

We used semiquantitative score systems to evaluate epidermal regeneration and granulation of slices. A four-point scale was used to evaluate granulation tissue formation (1, thin granulation layer; 2, moderate granulation layer; 3, thick granulation layer; 4, very thick granulation layer), and a three-point scale was used to evaluate dermal and epidermal regeneration (1, little regeneration; 2, moderate regeneration; 3, complete regeneration).

For immunohistochemistry, thick sections (5 μm) were reacted with anti-F4/80 primary antibody (1:200 dilution, ab111101, Abcam, Cambridge, MA, USA) or anti-TGF-β1 primary antibody (1:500, ab92486, Abcam, Cambridge, MA, USA) incubated overnight at room temperature after blocking endogenous peroxidase and nonspecific binding. After washing in PBS, the sections were incubated with biotinylated goat anti-rabbit IgG (1:200 dilution, ab150077; Abcam) for 1 h at room temperature. For the control group, sections were treated with the same dilution buffer without primary antibodies. Finally, the cells immunostained with anti-F4/80 antibody (n = 6/group) were counted to evaluate the infiltration of macrophages.

### Inflammatory assay

TNF-α and TGF-β1 were used to evaluate the immunomodulatory activity of samples, as per previous research^17^. For the cytokine secretion assay, the human monocyte-like cell line THP-1 (ATCC TIB-202) was cultured in RPMI 1640 medium with 10% FBS, 1% L-glutamine (Gibco, Carlsbad, CA, USA), 100 U/mL of streptomycin, and 100 U/mL of penicillin in a humidified atmosphere of 5% CO_2_ at 37 °C. The cells (2 × 10^5^/mL, 100 μl, respectively) were plated in 96-well plates and differentiated into adherent macrophage-like cells by the addition of phorbol myristate acetate for 3 d. Afterwards, samples at various concentrations (100 nM, 1 μM, and 10 μM) dissolved in serum-free medium with *E. coli* O111: B4 LPS (Sigma-Aldrich, St Louis, MO, USA) were added to each well. The same volume of serum-free medium was used as a blank control. For the other control, we added LPS alone to the medium without 10% FBS. After incubation for 12 h, the supernatants were collected to test the production of TNF-α and TGF-β1 using an ELISA kit following the manufacturer’s protocols.

### Hemolytic activity assay

The hemolytic activity of the samples was tested with healthy human erythrocytes in liquid medium, as described previously^31^. Healthy red blood cells were provided by the Kunming Blood Center. In the experiment, cells were twice washed with PBS and centrifuged at 3000 g for 5 min at room temperature, after which the red blood cells and samples were incubated in a water bath for 30 min at 37 °C. After centrifugation at 5000 g for 3 min at room temperature, supernatant absorbance was measured at 540 nm. Maximum hemolysis was determined by incubation with 1% Tritonx-100. Hemolytic activity (%) was calculated by (A_sample_) × 100/A_tritonx-100_.

### Acute toxicity assay

Acute toxicity was determined in two phases, as described previously^32^. Lethal range acute toxicity of the cathelicidin-OA1 test samples was determined at concentrations of 5, 10, 20, and 40 μmol/kg by i.p. injection in mice. Each group contained five male and five female mice. The mice were observed for two weeks, with mortalities, toxic effects, and behavioral changes recorded.

## Electronic supplementary material

supplemental data
