# Author Correction: Cathelicidin-OA1, a novel antioxidant peptide identified from an amphibian, accelerates skin wound healing

**DOI:** 10.1038/s41598-018-33558-w

**Published:** 2018-10-23

**Authors:** Xiaoqing Cao, Ying Wang, Chunyun Wu, Xiaojie Li, Zhe Fu, Meifeng Yang, Wenxin Bian, Siyuan Wang, Yongli Song, Jing Tang, Xinwang Yang

**Affiliations:** 1 Department of Pathology, Faculty of Basic Medical Science, Kunming Medical University, Kunming, 650500 Yunnan China; 2 Key Laboratory of Chemistry in Ethnic Medicine Resource, State Ethnic Affairs Commission & Ministry of Education, School of Ethnomedicine and Ethnopharmacy, Yunnan Minzu University, Kunming, 650500 Yunnan China; 3 Department of Anatomy and Histology & Embryology, Faculty of Basic Medical Science, Kunming Medical University, Kunming, 650500 Yunnan China; 4 Department of Biochemistry and Molecular Biology, Faculty of Basic Medical Science, Kunming Medical University, Kunming, 650500 Yunnan China

Correction to: *Scientific Reports* 10.1038/s41598-018-19486-9, published online 17 January 2018

This Article contains errors. Figure 8 was misassembled during the preparation of the manuscript: incorrect images were used for panel 8C, and for the vehicle image in panel 8E. The correct Figure 8 appears below as Figure 1.

**Figure 1:**
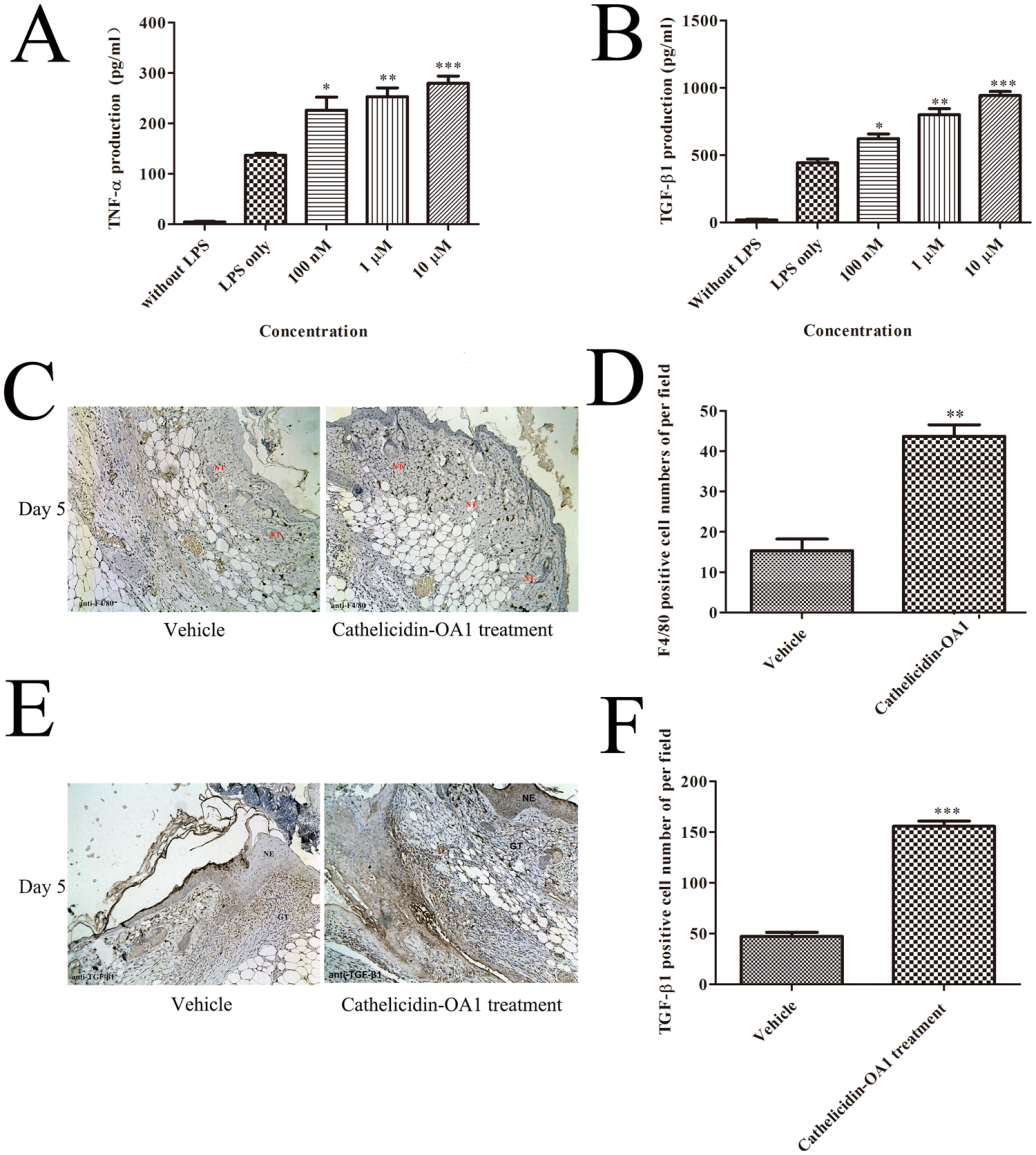
Cathelicidin-OA1 promoted the secretion of TNF-α and TGF-β, macrophages recruitment, TGF-β expression. (**A**,**B**) THP-1 cells were stimulated by LPS to secrete TNF-α and TGF-β1. Incubation with cathelicidin-OA1 at different concentrations resulted in the dose-dependent increase in TNF-α and TGF-β1 secretion. *P < 0.05, **P < 0.01, and ***P < 0.0001 indicate significantly different from the negative control (Student’s *t*-test). Data are mean values of three independent experiments performed in triplicate. (**C**,**E**) Immunohistochemical results for anti-F4/80 and TGF-β1. (**D**,**F**) F4/80 or TGF-β1 positive cell numbers per high power field were significantly different between cathelicidin-OA1 treatment and the control. **P < 0.01 and ***P < 0.0001. Data are mean values of three independent experiments performed in triplicate and six different fields for each section (×100).

The conclusions of the Article are unaffected by this correction.

