# Supplementary material for: Cathelicidin-OA1, a novel antioxidant peptide identified from an amphibian, accelerates skin wound healing
Source: Sci Rep. 2018 Jan 17;8:943. doi: 10.1038/s41598-018-19486-9 (PMC5772731; doi:10.1038/s41598-018-19486-9)
Supplement: Supplementary file 1 — supplemental data [file 41598_2018_19486_MOESM1_ESM.pdf]

Cathelicidin-OA1, a novel antioxidant peptide identified from an amphibian, accelerates skin wound healing.

Xiaoqing Cao[1,§], Ying Wang[2,§], Chunyun Wu[3,§], Xiaojie Li[4], Zhe Fu[3], Meifeng Yang[3], Wenxin Bian[3], Siyuan Wang[2], Yongli Song[3], Jing Tang[4,*], Xinwang Yang[3,*]

1. Department of Pathology, Faculty of Basic Medical Science, Kunming Medical University, Kunming 650500, Yunnan, China.

2. Ethic Drug Screening & Pharmacology Center, Key Laboratory of Chemistry in Ethnic Medicine Resource, State Ethnic Affairs Commission & Ministry of Education, Yunnan Minzu University, Kunming 650500, Yunnan, China.

3. Department of Anatomy and Histology & Embryology, Faculty of Basic Medical Science, Kunming Medical University, Kunming 650500, Yunnan, China.

4. Department of Biochemistry and Molecular Biology, Faculty of Basic Medical Science, Kunming Medical University, Kunming 650500, Yunnan, China.

[§]These authors contributed equally to this work

[*]Corresponding authors:

Dr. Xinwang Yang, Faculty of Basic Medical Science, Kunming Medical University, 1168 West Chunrong Road, Kunming, Yunnan, 650500. Tel: +86 13577174345. Email: yangxinwanghp@163.com

Dr. Jing Tang, Faculty of Basic Medical Science, Kunming Medical University, 1168 West Chunrong Road, Kunming, Yunnan, 650500. Tel: +86 13629600409. Email: gracett916@163.com

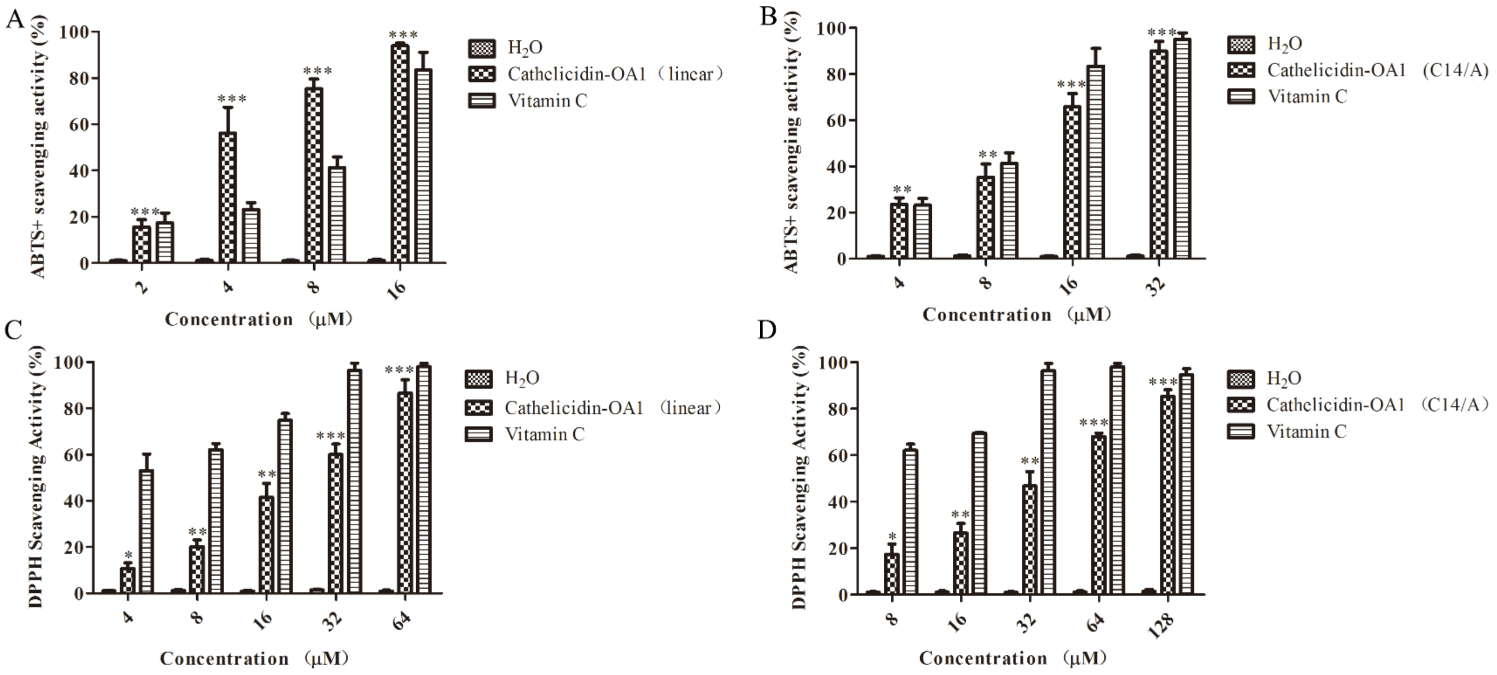

Figure S1. The antioxidant activities of cathelicidian-OA1 (C14/A) and cathelicidian-OA1 (linear).

(A) Dose-dependent $ABTS^+$ free radical scavenging activity of linear cathelicidin-OA1. (B) Dose-dependent $ABTS^+$ free radical scavenging activity of cathelicidin-OA1 (C14/A). (C) Dose-dependent DPPH free radical scavenging activity of linear cathelicidin-OA1. (D) Dose-dependent DPPH free radical scavenging activity of cathelicidin-OA1 (C14/A). 'H_2O' indicates 'negative control' and 'vitamin C' indicates 'positive control'. *$P < 0.05$, **$P < 0.01$, and ***$P < 0.0001$ indicate significant difference from the negative control (Students *t*-test). Data are mean values of three independent experiments performed in triplicate

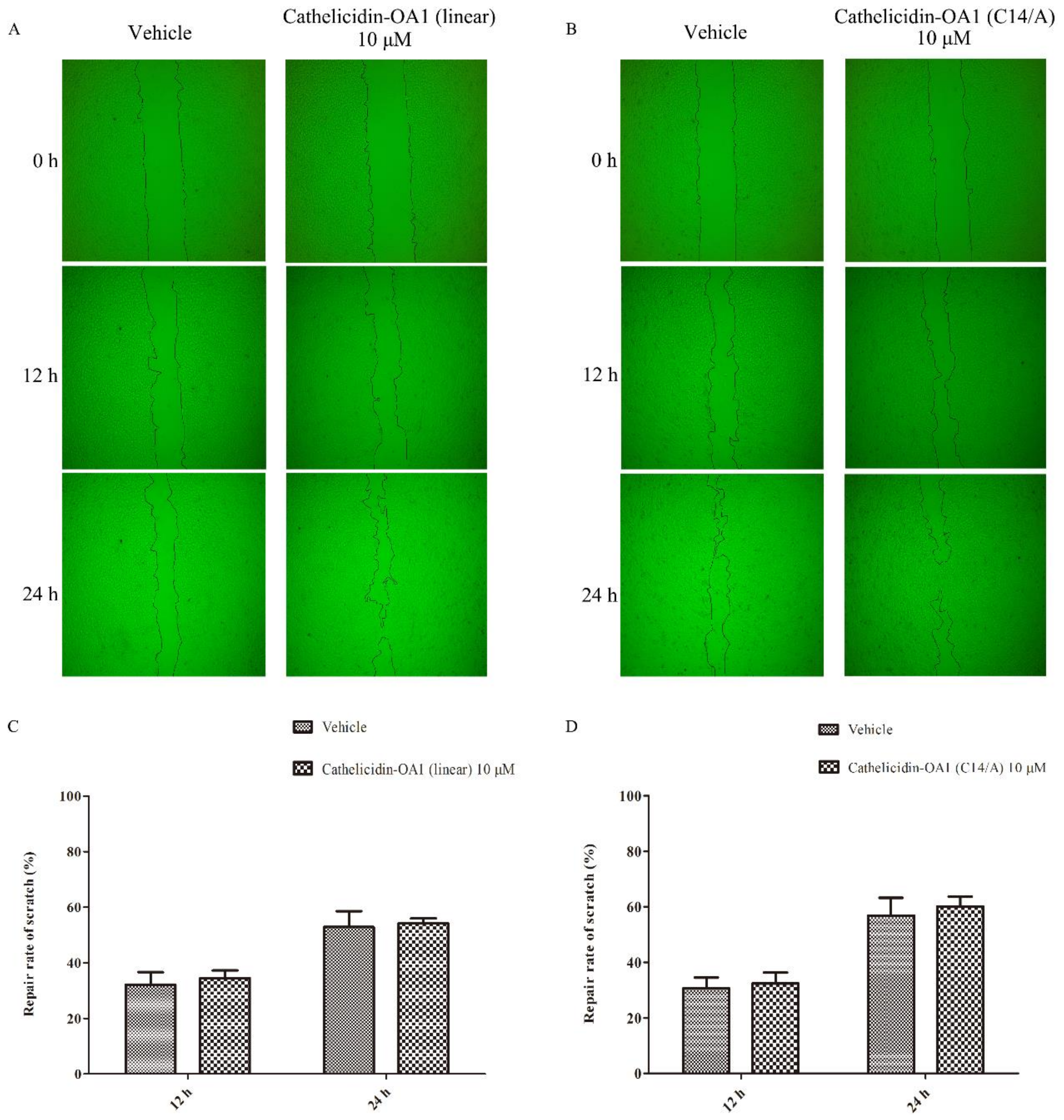

Figure S2. The effects of cathelicidin-OA1 (C14/A) and cathelicidian-OA1 (linear) on the HaCaT cell wounds (scratches).

(A, C) Cathelicidin-OA1 (linear) (10 μM) showed no obvious HaCaT cell wound-healing activity. (B, D) Cathelicidin-OA1 (C14/A) (10 μM) illustrated no obvious HaCaT cell wound-healing activity. 'Vehicle' is the negative control; Data are mean values of three independent experiments performed in triplicate.

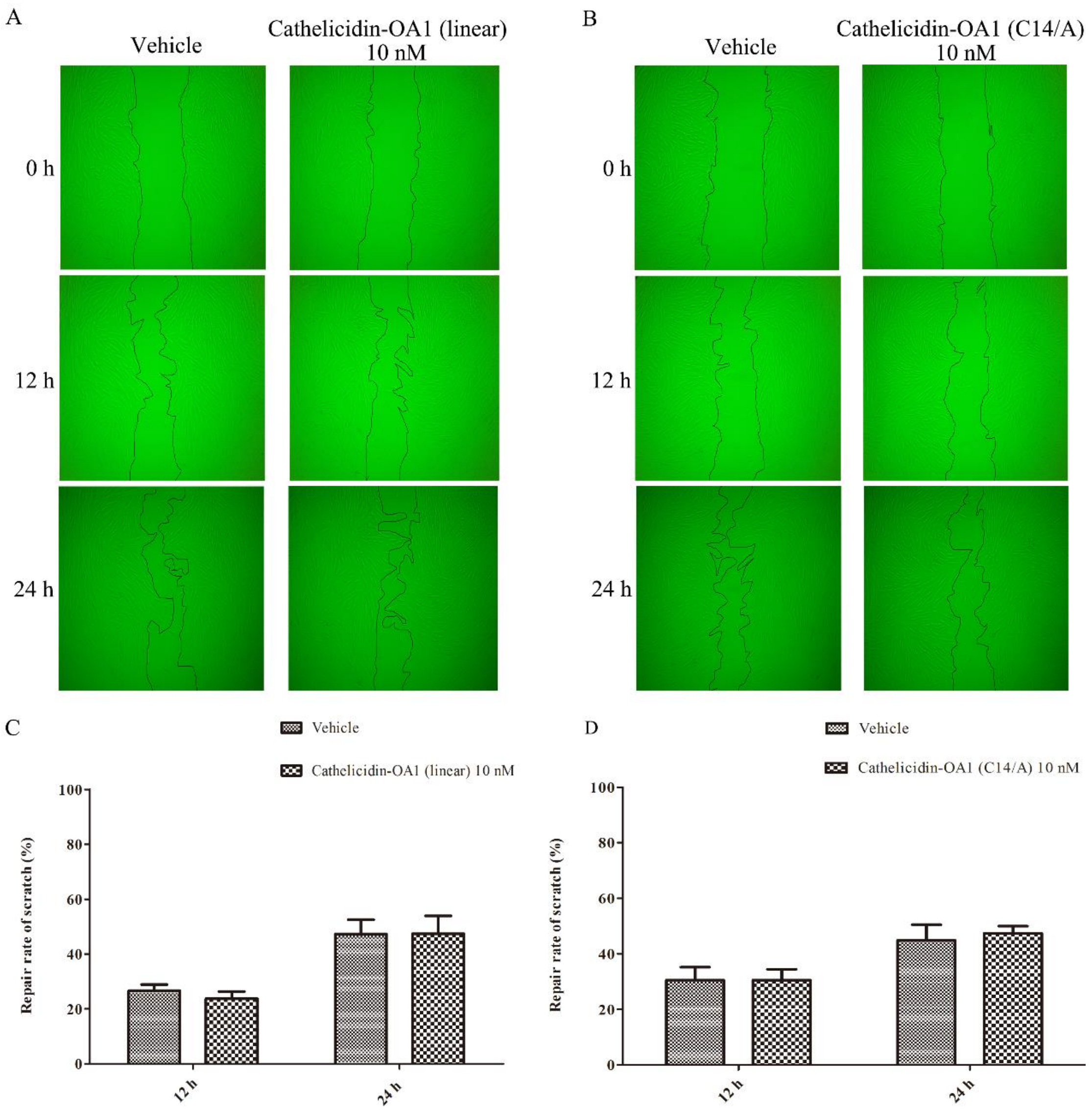

Figure S3. The effects of cathelicidin-OA1 (C14/A) and cathelicidian-OA1 (linear) on the HSF ell wounds (scratches).

(A, C) Cathelicidin-OA1 (linear) (10 nM) showed no obvious HSF cell wound-healing activity. (B, D) Cathelicidin-OA1 (C14/A) (10 nM) illustrated no obvious HSF cell wound-healing activity. 'Vehicle' is the negative control; Data are mean values of three independent experiments performed in triplicate.
